# Virological Sampling of Inaccessible Wildlife with Drones

**DOI:** 10.3390/v10060300

**Published:** 2018-06-02

**Authors:** Jemma L. Geoghegan, Vanessa Pirotta, Erin Harvey, Alastair Smith, Jan P. Buchmann, Martin Ostrowski, John-Sebastian Eden, Robert Harcourt, Edward C. Holmes

**Affiliations:** 1 Department of Biological Sciences, Macquarie University, Sydney, NSW 2109, Australia; vanessa.pirotta@hdr.mq.edu.au (V.P.); robert.harcourt@mq.edu.au (R.H.); 2 Marie Bashir Institute for Infectious Diseases and Biosecurity, Charles Perkins Centre, School of Life and Environmental Sciences and Sydney Medical School, The University of Sydney, Sydney, NSW 2006, Australia; ehar6487@uni.sydney.edu.au (E.H.); jan.buchmann@sydney.edu.au (J.P.B.); js.eden@sydney.edu.au (J.-S.E.); edward.holmes@sydney.edu.au (E.C.H.); 3 Heliguy Scientific Pty Ltd., Sydney, NSW 2204, Australia; alastair@heliguy.tv; 4 Department of Molecular Sciences, Macquarie University, Sydney, NSW 2109, Australia; martin.ostrowski@mq.edu.au; 5 Westmead Institute for Medical Research, Centre for Virus Research, Westmead, NSW 2145, Australia

**Keywords:** whale, virome, drone, mammalian host, virosphere

## Abstract

There is growing interest in characterizing the viromes of diverse mammalian species, particularly in the context of disease emergence. However, little is known about virome diversity in aquatic mammals, in part due to difficulties in sampling. We characterized the virome of the exhaled breath (or blow) of the Eastern Australian humpback whale (*Megaptera novaeangliae*). To achieve an unbiased survey of virome diversity, a meta-transcriptomic analysis was performed on 19 pooled whale blow samples collected via a purpose-built Unmanned Aerial Vehicle (UAV, or drone) approximately 3 km off the coast of Sydney, Australia during the 2017 winter annual northward migration from Antarctica to northern Australia. To our knowledge, this is the first time that UAVs have been used to sample viruses. Despite the relatively small number of animals surveyed in this initial study, we identified six novel virus species from five viral families. This work demonstrates the potential of UAVs in studies of virus disease, diversity, and evolution.

There is a growing interest in understanding the diversity, evolution, and disease associations of viruses in natural populations [1]. Although sampling of many terrestrial species is relatively straightforward, there may be serious logistical challenges for animals that live in inaccessible habitats. Marine environments are one such habitat [2,3,4]. It has recently been shown that wild populations can be sampled using Unmanned Aerial Vehicles (UAVs) [5,6]. UAVs are rapidly transforming wildlife science, allowing sampling from dangerous and inaccessible environments to address questions previously only approached by theory. Here, we show how UAVs can be used to sample viruses. This approach may ultimately enable a better understanding of the patterns and drivers of disease emergence in wild populations.

There is evidence that marine mammal health is deteriorating as anthropogenic stressors on the world’s oceans increase [7]. However, contemporary assessments of marine mammal health are strongly biased towards animals whose health is already compromised, such as stranded animals, which in part reflects the difficulties in sampling aquatic environments. Sampling from free-ranging marine mammals is therefore critical to assess whether healthy animal populations are potential reservoirs of viruses and other transmittable agents.

Following the use of UAV technology for sampling, we employed a meta-transcriptomic approach [8,9] to help characterize the virome of an important marine mammal, the Eastern Australian humpback whale (*Megaptera novaeangliae*), which serves as a model for work in this area. Recent analyses of whale breath, or “blow”, have revealed an extraordinary diversity and abundance of microbiota. Importantly, the microbial communities observed were divergent from those present in the surrounding seawater such that they could be considered as distinctly whale blow associated [5,6]. To date, however, these studies have not included virus sampling, and little is known about the diversity of the whale virome and whether this differs fundamentally from that seen in terrestrial mammals.

We collected whale blow samples from 19 humpbacks during the 2017 annual northward migration from Antarctica to northern Australia (Figure 1a). To adhere to all Australian legislative requirements, our UAVs were registered with the Civil Aviation Safety Authority (CASA) and operated by a CASA-certified remote pilot. All flights were conducted in good weather (no rain, Beaufort < 3), from a small research vessel, where the UAV was launched and landed on a launch pad at the stern of the boat. A closed, sterile petri dish was placed on eight suction cups on the UAV before each flight.

Members of the team visually scanned the area for humpback whales. Once an individual or pod was chosen, the vessel was driven at a constant speed and distance from the whale. Once the respiratory rhythm was determined (i.e., downtime length), the UAV was launched to coincide with surfacing. The UAV pilot was directed by spotters on the vessel and positioned the UAV with the aid of the live feed from a forward-facing camera. To minimize sample contamination, the petri dish remained closed until immediately before the whale surfaced. The dish was remotely opened as the UAV accelerated towards and through the densest part of the whale blow, collecting the maximum amount of sample in the dish and lid (see Video S1). The petri dish was immediately closed and the UAV was returned to the vessel. The petri dish containing the sample was removed from the UAV and secured with Parafilm^®^. All samples were stored immediately in a portable −80 °C freezer. A different whale was sampled each flight. Different individuals within a pod were chosen based upon unique distinctive markings (e.g., white flanks and barnacle arrangements).

RNA was extracted using an RNeasy Plus Universal mini kit (Qiagen, Australia). Due to low RNA concentration, all 19 samples were pooled and concentrated using a NucleoSpin RNA Clean-up XS kit (Macherey-Nagel, Australia). A single library was produced for RNA sequencing using the Low-Input SMARTer Stranded Total RNA Sample Prep Kit with Mammalian rRNA depletion (Clontech, Australia), with 1 ng of the pooled whale blow RNA as input. Paired-end (100  bp) sequencing of the RNA library was performed on the HiSeq 2500 platform (Illumina, Australia) at the Australian Genome Research Facility.

RNA sequencing of the rRNA-depleted library resulted in 19,389,378 paired reads (100 nt in length) that were assembled de novo into 107,681 contigs. Sequencing reads were first quality trimmed then assembled using Trinity [10]. The assembled transcriptome was annotated based on similarity searches against the NCBI nucleotide (nt) and non-redundant protein (nr) databases using BLASTn [11] and Diamond (BLASTX) [12], respectively, and an e-value threshold of 1 × 10^−5^. Transcript abundance was estimated using RSEM [13] implemented within Trinity.

Our transcriptome data revealed that the humpback whale blow contains a wide diversity of DNA and RNA viruses (that we refer to “whale-blow-associated” viruses). BLAST analysis revealed the relative abundance of taxonomic classes present in the non-rRNA transcriptome data, of which bacteria occupied ~45%, while ciliates were the second-most abundant source at ~29%. Importantly, Baleen whale species contributed 0.9% of the transcriptome data and were the most abundant source of mammalian RNA, indicating our sample is indeed whale associated. Viruses occupied ~0.01% of the non-rRNA transcriptome, which falls within the range of other meta-transcriptome studies of vertebrates [9]. Despite this relatively low abundance, the viral contigs observed fell into 42 classified viral families, including 29 families of bacteriophage (Figure 1b). The most relatively abundant bacteriophages included the *Siphoviridae* (18.4% of all viruses) and the *Myoviridae* (15.2% of all viruses). Among the most abundant viral families that are known to infect eukaryotes were small single-stranded (ss) DNA viruses, specifically the *Circoviridae* (and *Circoviridae*-like viruses) (6.5% of all viruses), as well as members of the *Parvoviridae* (2.4%) and an RNA virus family, the *Tombusviridae* (0.9%).

We next inferred the evolutionary relationships of the viruses contained in whale blow with their closest phylogenetic relatives. Translated open reading frame segments were combined with protein sequences obtained from GenBank, using the top search results from BLAST (see Table 1 for more details of the sequences analyzed). Sequences were aligned using MAFFT v.3.4 [14], employing the E-INS-I algorithm with poorly aligned regions removed using trimAl v.1.2 [15]. To estimate phylogenetic trees for the virus data sets, we selected the optimal amino acid substitution model identified using the Bayesian Information Criterion as implemented in Modelgenerator v.0.85 [16] and analyzed the data using the maximum likelihood approach available in PhyML v3.1 [17] with 1000 bootstrap replicates. Phylogenetic trees were annotated with FigTree v.1.4.2.

Of the most abundant eukaryotic viruses, two novel (as determined by phylogenetic analysis) circular Rep-encoding ssDNA viruses (CRESS-DNA viruses) *Circoviridae*-like viruses were identified, denoted here as humpback whale blow-associated circo-like virus 1 and 2 (Table 1; Figure 2). Related viruses have previously been identified in many aquatic systems, for which marine invertebrates, particularly crustaceans, are thought to be a primary host [18]. Humpback whale blow-associated circo-like virus-1 exhibited 51% amino acid identity to the replication-associated protein (Rep) of its closest genetic relative, sewage-associated circular DNA virus-29, and 46% amino acid identity to the Rep of Lake Sarah associated circular virus-32. Humpback whale blow-associated circo-like virus-2 shared 46% amino acid identity to the Rep of McMurdo Ice Shelf virus-5, isolated from a freshwater pond in Antarctica [19]. As these ssDNA viruses appear to be major virome components in many aquatic environments [18], they are likely associated with aquatic ecosystems in general.

Another relatively abundant viral contig was a partial genome of a novel densovirus (family *Parvoviridae*). The most similar amino acid sequence to this new virus, denoted here as humpback whale blow-associated denso-like virus, was a densovirus isolated from a *Periplaneta fuliginosa* (i.e., a cockroach), sharing only 47% sequence similarity to the nonstructural protein (Table 1; Figure 2). Similarly, a novel tombus-like viral partial genome, falling into the *Tombusviridae*, was identified and was closely related to Changjiang tombus-like virus-9 isolated from crayfish, with 41% sequence similarity to the RNA-dependent RNA polymerase (RdRp). We denote this virus humpback whale blow-associated tombus-like virus (Table 1; Figure 2).

To reveal viruses at very low relative abundance, a Diamond BLAST [12] analysis was performed against the raw 100 bp sequencing reads. This process identified several sequencing reads that matched viruses, later assembled into short contigs, that comprised two potentially new RNA viruses from the *Picornaviridae* and the *Astroviridae*. Humpback whale blow-associated picornavirus shared 61% amino acid similarity to the RdRp of the most closely related *Coturnix coturnix* (quail) picornavirus (Table 1; Figure 2). Similarly, humpback whale blow-associated astrovirus shared 76% amino acid identity with the nonstructural protein 1a of porcine astrovirus-5 (Figure 2). Both picornaviruses and astroviruses are single-stranded, positive-sense RNA viruses with small icosahedral capsids and no external envelope which may aid their preservation in harsh marine environments, and viruses from these families are commonly found in aquatic vertebrates [9]. As only short fragments of these viruses’ genomes were identified in our data set, their phylogenetic position requires confirmation. This is likely due to the low quantity of RNA isolated from the whale blow samples and the pooling of individual samples. However, that both these viruses were most closely related to other vertebrate viruses suggested that they are likely whale associated rather than sampled from the surrounding seawater. Further sampling of the sea water virome is required to understand the potential enormous diversity that comprises the aquatic virosphere.

Little is known about the transmission of whale viruses. Analyses of whale influenza viruses suggest that they likely originated from gulls and that feeding activities of gulls and whales often place them in close contact, such that oral–fecal transmission through seawater is a likely route [20] and which might explain our observation of viruses associated with aquatic ecosystems. In addition, given the vast aerosol produced by whales, and their close contact within migrating pods as well as at feeding and breeding grounds, respiratory transmission may also play an important role in the movement of viruses in whales. 

In sum, we show that drone-based virological surveys of previously inaccessible wildlife populations has the potential to help reveal the diversity of the virosphere, facilitating the detection of viruses infecting wildlife and aiding evaluation of their pathogenic and zoonotic potential.

## Figures and Tables

**Figure 1:**
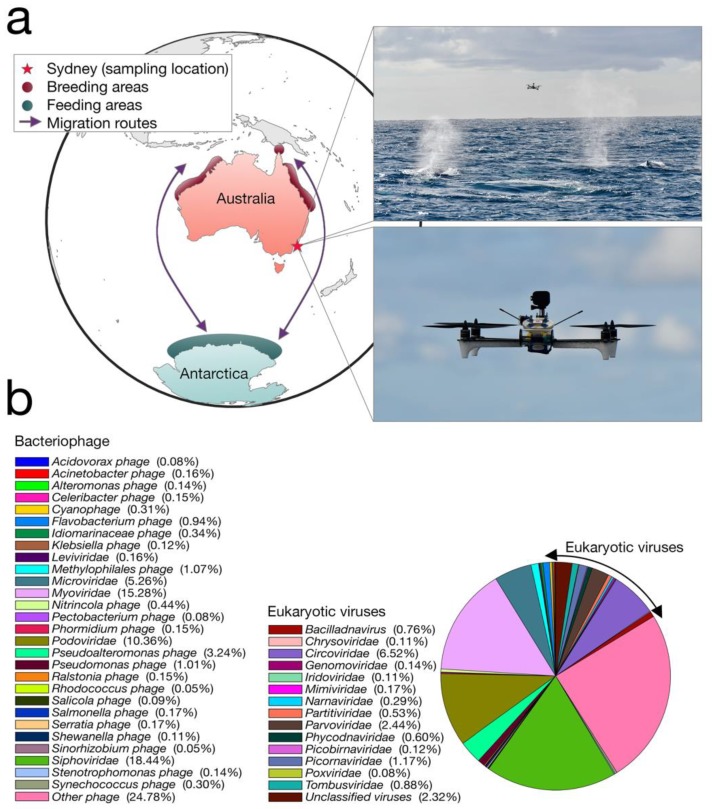
(**a**) Map showing the humpback whale sampling location (red star), approximately 3 km off the coast of Sydney, New South Wales, Australia. Purple arrows indicate the typical seasonal migratory routes of the humpback whale from their likely feeding ground in Antarctica (dark green) to their breeding areas around northern Australia (dark red). Photographs demonstrate the Unmanned Aerial Vehicle (UAV) in action. (**b**) Relative abundance of viruses and their taxonomic families. Taxonomy was based on both protein and nucleotide BLAST search results, taking the best e-value for each (for those with identical e-values, we used the taxa with the closest percentage identity). This included 42 viral families, including 29 families of bacteriophage. Percentages indicate relative abundance of all viruses in the sequence library.

**Figure 2:**
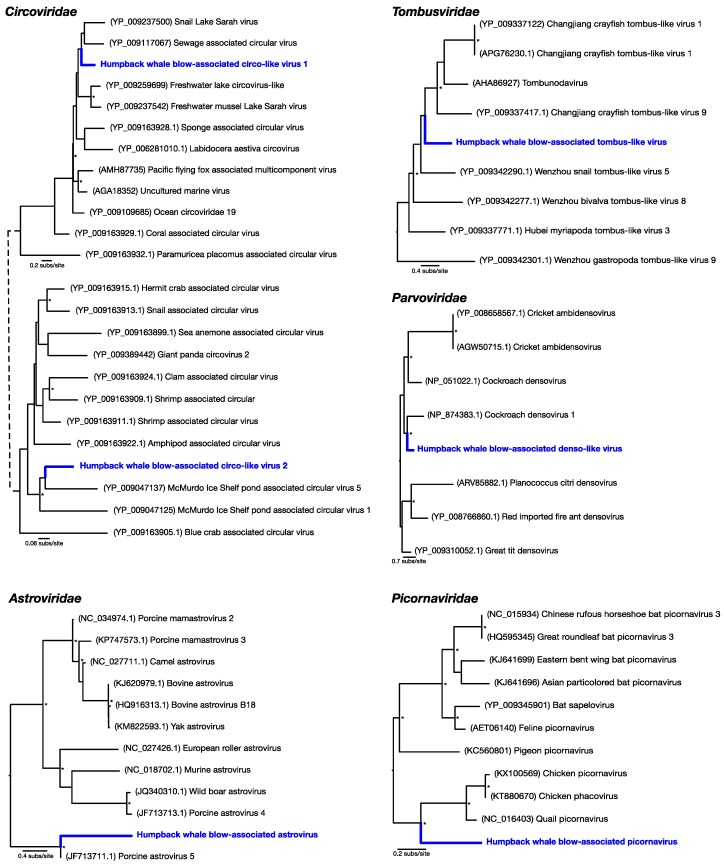
Phylogenetic relationships of the viruses discovered from assembled contigs along with their closest genetic relatives obtained from GenBank (accession numbers in parentheses). The families described here are *Circoviridae*-like, *Parvoviridae*, *Tombusviridae*, *Picornaviridae*, and *Astroviridae*. The maximum likelihood phylogenetic trees show the topological position of the newly discovered viruses (blue). Asterisks indicate branch support >70%, based on 1000 bootstrap replicates. All branches are scaled per the number of amino acid substitutions per site. Trees were midpoint rooted for clarity only.

**Table 1:** Amino acid identity, contig length, and relative frequency of the viruses identified in this study. All sequence reads generated in this project are available under the NCBI Short Read Archive (SRA) under accession number SRP149185 and virus sequences have been deposited in GenBank.

Virus Family	Virus Species	Contig Length (nt)	% Relative Abundance in Library	% Amino Acid Identify	Closest Match (GenBank Accession Number)
*Circoviridae*	Humpback whale blow-associated circo-like virus 1	702	0.000115%	51%	Sewage-associated circular DNA virus-29 (YP_009117067)
*Circoviridae*	Humpback whale blow-associated circo-like virus 2	909	0.000164%	46%	McMurdo Ice Shelf pond-associated circular DNA virus-5 (YP_009047137)
*Parvoviridae*	Humpback whale blow-associated denso-like virus	315	0.000143%	47%	*Periplaneta fuliginosa* densovirus (NP_051022.1)
*Tombusviridae*	Humpback whale blow-associated tombus-like virus	279	0.000164%	41%	Changjiang tombus-like virus-9 (YP_009337417.1)
*Picornaviridae*	Humpback whale blow-associated picornavirus	255	(N/A–assembled contigs from raw reads)	61%	Quail picornavirus (NC_016403)
*Astroviridae*	Humpback whale blow-associated astrovirus	130	(N/A–assembled contigs from raw reads)	76%	Porcine astrovirus 5 (YP_009010969)

## Supplementary Materials

The following are available online at http://www.mdpi.com/1999-4915/10/6/300/s1. Video S1: GoPro footage from UAV demonstrates whale blow sampling. All sequence reads generated in this project are available under the NCBI Short Read Archive (SRA) under accession number SRP149185 and virus sequences have been deposited in GenBank.
